# Reaching people who do not use eye services

**Published:** 2014

People with eye problems in India, Nepal and the Gambia gave the following as their main reasons for not seeking treatment:

- fear (that surgery will damage or ‘spoil’ the eyes, or miscellaneous fears)
- inability to leave family or work responsibilities
- put off by the post-operative recommendations
- treatment cost
- feel they can manage – that treatment is not necessary
- too old
- fatalistic – ‘God's will’
- no-one to accompany them
- distance and lack of transport

Despite the differences in geographical and cultural settings, there was a remarkable consensus of opinion amongst people about why they did not seek treatment.

Providers tend to attribute poor user demand to a lack of awareness of treatment availability and benefit. Lack of knowledge or understanding may explain a proportion of non-use of eye services but it is not the root cause. It is known that poor service use occurs also amongst communities with a good knowledge of eye problems and treatment options.

Another commonly held view is that people need to be motivated to seek treatment. Individuals are motivated, but their motivations may differ from that of the provider community. When viewed in context, many of the reasons given above start to make sense.

## 1 Fear

The fear that treatment such as cataract surgery will ‘spoil’ eyes may not be irrational. In response to concerns about the quality of cataract surgical outcomes, the World Health Organization (WHO) strongly recommends the need for better monitoring and evaluation systems. It is well known that ‘bad news travels fast’. Treatment failures may – unfortunately – impact more upon community attitudes to eye treatment than all the examples of success.

## 2 Cost in time and money

Dealing with direct treatment costs has been a major concern of service providers, and is a very important obstacle to overcome. However, these are only part of the cost borne by service users and their families. The concept of ‘time is money’ is not only the preserve of the city professional. In fact it has a sharper reality for people living in poverty. Seeking treatment involves leaving day-to-day responsibilities. In an existence of ‘work today, eat today’ early treatment is a luxury that may be unaffordable. Costs are multiplied when other family members are involved, either to fulfil roles as carers or to accompany the person for surgery.

**Figure:**
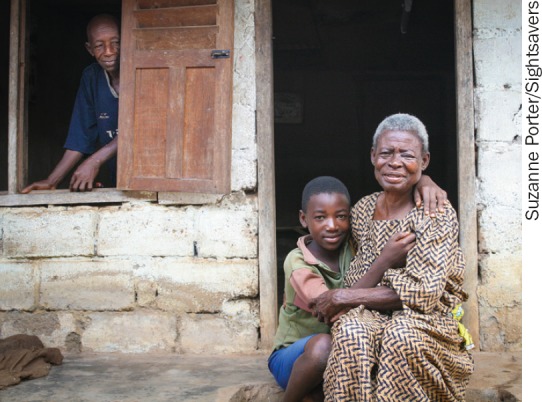
Many older people accept poor eyesight

## 3 Attitudes to old age and gender

Unless actively addressed, there is scope for negative attitudes to old age and female gender to become a bigger barrier to treatment. Cataract is an age-related condition. Given demographic forecasts and life expectancy patterns, many of the people requiring surgical treatment will also be women (including widows). In many communities these are the people who are likely to be forgotten.

## 4 ‘I don't need treatment – I can manage’

To a greater or lesser extent, people report that they are coping and do not perceive a need for treatment or surgery. This includes people who are blind in both eyes too. This is somewhat surprising but a possible explanation is that they have adjusted to their disability. On the other hand, this response may mask hidden barriers. After weighing up the advantages and disadvantages it is not worth the bother – ‘I'll manage’. Currently the explanation is not clear, and requires further exploration.

## Conclusion

We need to raise awareness about the low use of cataract services, and adopt strategies that promote equality in eye service delivery, access and use. People who do not use eye services know why they do not seek treatment. It is therefore critical that providers ask and listen to the views of their community.

A précis of an article written by Martine Donaghue in the Community Eye Health Journal, Volume 12 No. 31, 1999.

